# Cytochrome P450 Sterol 14 Alpha-Demethylase Gene *SsCI72380* Is Required for Mating/Filamentation and Pathogenicity in *Sporisorium scitamineum*

**DOI:** 10.3389/fmicb.2021.696117

**Published:** 2021-12-23

**Authors:** Huizhong Li, Yichang Cai, Quanqing Deng, Han Bao, Jianwen Chen, Wankuan Shen

**Affiliations:** ^1^College of Agriculture, South China Agricultural University, Guangzhou, China; ^2^Sugarcane Research Laboratory, South China Agricultural University, Guangzhou, China; ^3^Scientific Observing and Experimental Station of Crop Cultivation in South China, Ministry of Agriculture and Rural Areas, Guangzhou, China

**Keywords:** *Sporisorium scitamineum*, cytochrome P450 sterol 14 alpha-demethylase, ergosterol, sexual mating, pathogenicity

## Abstract

Sugarcane smut is a significant sugarcane disease caused by *Sporisorium scitamineum* and is a large threat to the sugar industry in China and the world. Accordingly, it is important to study the pathogenic mechanism by which this disease occurs to identify effective prevention and control strategies. Gene *SsCI72380*, which encodes cytochrome P450 sterol 14 alpha-demethylase (CYP51), was screened out from the transcriptome of *S. scitamineum*. In this study, the functions of gene *SsCI72380* were identified *via* the knockout mutants *ΔSs72380^+^* and *ΔSs72380^−^*, which were obtained by polyethylene glycol (PEG)-mediated protoplast transformation technology, as well as the complementary mutants *COM72380^+^* and *COM72380^−^*. The results showed that the CYP51 gene *SsCI72380* played an important role in sporidial growth, sexual mating/filamentation, hyphae growth, and pathogenicity in *S. scitamineum*. Gene *SsCI72380* may regulate the biosynthesis process of ergosterol by encoding CYP51 enzymes and then affecting the structure and function of the cell membrane. Gene *SsCI72380* also played an important role in the response toward different abiotic stresses, including hyperosmotic stress, oxidative stress, and cell wall stress, by regulating the permeability of the cell membrane. In addition, gene *SsCI72380* is a new type of pathogenic gene from *S. scitamineum* that enhances the pathogenicity of *S. scitamineum*.

## Introduction

Sugarcane smut causes serious economic losses in sugarcane globally and is caused by *Sporisorium scitamineum*, which belongs to the Basidiomycetes subphylum Melanophila (Nzioki et al., 2010). *Sporisorium scitamineum* is a two-type fungus. Its life history can be divided into two stages: a stage of a yeast-like basidiospore without pathogenicity, and another stage of a dikaryotic hypha with pathogenicity (Bakkeren et al., 2008; Que et al., 2014). When the teliospore of *S. scitamineum* infects sugarcane (*via* sugarcane bud invasion), the pathogenic dikaryotic hypha grows together with the young bud meristem of the sugarcane, and the appressorium grows on the young bud scales of the sugarcane. With the fusion of the two compatible nuclei of the dikaryotic hypha, diploid teliospores are formed, and the sugarcane plants produce black whip symptoms (Taniguti et al., 2015). The teliospores are nearly round, 5–6 μm in diameter, reddish brown or black in color, and can germinate promycelia of different lengths under suitable temperature and humidity conditions. Each promycelia produces four oval and transparent basidiospores, of which two are “+” mating haploids and two are “−” mating haploids. Compatible mating haploids form pathogenic dikaryotic hypha through sexual mating. Therefore, the pathogenicity of *S. scitamineum* is closely related to its sexual mating and subsequent filamentation (Albert and Schenck, 1996; Chen et al., 2015).

*Sporisorium scitamineum* is a dimorphic fungus, and the transformation from haploid to diploid is not only a morphological change from yeast-like to mycelium but also an important genetic change (Que et al., 2014). Based on homologous recombination gene knockout technology, Yan et al. (2016) showed that the gene *b*E of the locus *b* of *S. scitamineum* is related to the sexual mating and pathogenicity of this pathogen. After the gene was knocked out, the sexual mating ability of WT17 and WT18 was completely lost, and they were unable to infect sugarcane. Chang et al. (2018) found that the cAMP/PKA signaling pathway can regulate the intracellular reactive oxygen species (ROS) level by limiting the transcription of metabolic enzymes and then regulating the sexual mating ability of *S. scitamineum*. Deng et al. (2018b) found that the *SsKpp2* gene encoding mitogen-activated protein kinase (MAPK) in *S. scitamineum* affected the sexual mating and mycelial formation of the fungus by regulating tryptophan biosynthesis and the pheromone signal transduction pathway. Zhang et al. (2019) identified an autophagy gene *ATG8* in *S. scitamineum*. The deletion of the *ATG8* gene caused the single haploid sporidia of *S. scitamineum* to be pseudomycelium-like and sensitive to oxidative stress. Based on gene knockout and gene complementation techniques, Sun et al. (2019) found that the gene *Ram1* encoding the β-subunit of farnesyl transferase in *S. scitamineum* regulates the sexual mating, pathogenicity, and cell wall stability of the fungus. Wang et al. (2019) found that the gene *SsAgc1* encoding AGC kinase in *S. scitamineum* is related to the sexual mating and mycelial formation of the fungus, and further inferred that the function of the gene may be related to the synthesis of the small-molecule signal substance tryptophan. In addition, Zhu et al. (2019) reported that the pheromone response factor *SsPRF1* was involved in the regulation of sexual mating, mycelial growth, and pathogenicity of the fungus.

Sterol 14α-demethylase is a member of the oldest cytochrome P450 monooxygenase family commonly found in fungi/yeasts, higher plants, and mammals (Yuzo et al., 2000). It is involved in the biosynthesis of fungal ergosterols, phytosterols, and mammalian cholesterol. It is also a target of many azole antifungal drugs (Michael and Galina, 2005; Galina and Michael, 2006). The lack or complete absence of ergosterol biosynthesis will alter the fluidity of the fungal cell membrane and might change the activities of related enzymes on the fungal cell membrane, affecting the function of the fungal cell membrane and inhibiting the growth of fungi (Daum et al., 1998; Lees et al., 1999; Minnebruggen et al., 2010; Felipe et al., 2020). In the study of antifungal mechanism of cinnamaldehyde, under the action of cinnamaldehyde, the expression of the *ERG11* gene encoding sterol 14 α-demethylase in *Fusarium sambucinum* was downregulated, and the ergosterol content in this fungus was decreased by 67.94%, which resulted in cell membrane damage, a slow spore growth rate, and a decrease in pathogenicity (Wei et al., 2020). In the study of antifungal mechanism of *Euphorbia humifusa*, after treatment with *E. humifusa* extract (containing 40% flavonoids and 16% tannic acid), the activity of sterol 14 α-demethylase on the cell membrane of *Trichophyton rubrum* decreased, which affected ergosterol biosynthesis and inhibited the growth of *T. rubrum* (Li et al., 2014b). The *ERG11* gene encoding sterol 14 α-demethylase in the *Saccharomyces cerevisiae* Y12667 strain is located on chromosome VIII, and its deletion reduced the growth rate of the strain (Chen et al., 2009). However, no studies have been reported on the related genes encoding sterol 14α-demethylase in *S. scitamineum*.

Based on the previous transcriptome sequencing data of two *S. scitamineum* isolates *Ss*16 and *Ss*47 with different pathogenicities in our laboratory (Wu et al., 2020), a gene, *SsCI72380* (GenBank accession no. MZ004860), with a conserved structure encoding sterol 14α-demethylase of the cytochrome P450 family, was screened from the highly pathogenic isolate *Ss*16 and exhibited a significantly upregulated expression level. The purpose of this study was to explore the biological function of the gene *SsCI72380* in *S. scitamineum* by target gene knockout, gene complementation, phenotype analysis of gene deletion mutants and complements, and pathogenicity identification.

## Materials and Methods

### Characterization of the *SsCI72380* Gene Sequence

Based on transcriptome sequencing data, a gene *SsCI72380* encoding sterol 14α-demethylase was identified as a significantly (*p* ≤ 0.05) differentially expressed gene in isolates *Ss16* (strong pathogenicity) and *Ss47* (weak pathogenicity) of *S. scitamineum* with different pathogenicities (Wu et al., 2020). The protein sequence of *SsCI72380* was analyzed using the Compute pI/MW tool^1^ to determine the theoretical isoelectric point (pI) and molecular weight (MW). Blast comparison based on the amino acid sequences (DNA sequences into amino acid sequences)^2^ was performed on the NCBI database^3^ to obtain the conserved domain of the protein encoded by the *SsCI72380* gene. Phylogenetic analysis of the protein encoded by the *SsCI72380* gene was carried out in MEGA 7, and the phylogenetic tree was drawn using the neighbor-joining method (Saitou and Nei, 1987; Kumar et al., 2016). The Predotar online tool^4^ was used to predict the subcellular localization.

### Fungal Isolates and Culture Conditions Used in This Study

The wild-type haploid isolates *Ss16^+^* and *Ss16^−^* of *S. scitamineum* were isolated and identified in our laboratory and stored at −80°C (Deng et al., 2018a). The culture medium used in this study included YePSA medium (yeast extract 1%, peptone 2%, sucrose 2%, and agar 2%) and YePS liquid medium (yeast extraction 1%, peptone 2%, sucrose 2%, and pH 7.0), YePS soft medium (yeast extract 1%, peptone 2%, sugar 2%, and agar 0.65%) and YePSS medium (yeast extract 1%, peptone 2%, sugar 2%, D-sorbitol 18.17%, and agar 2%). For the mating/filamentation assay, equal volumes of wild-type, deletion mutant, or complementary mutant haploid sporidia of opposite mating-types were mixed and plated on solid medium in the absence or presence of 5 mM cAMP and 0.02 mM tryptophol and then kept in the dark in a 28°C incubator for 42 h before photographing. For stress tolerance assessment, the sporidial culture at OD600 = 1.0 and its 10-fold serial dilutions were inoculated on YePSA medium in the absence or presence of stress inducers, including 100 μg/ml Congo red (CR), 50 μg/ml SDS, 4 mM H_2_O_2_, and 500 mM NaCl, and then incubated in the dark at 28°C for 48 h before assessment and photographing. For the growth assay, sporidia of *S. scitamineum* wild-type (*Ss16^+^* and *Ss16^−^*), deletion mutant (*ΔSs72380^+^* and *ΔSs72380^−^*), and complementary mutant (*COM72380^+^* and *COM72380^−^*) were cultured in 50 ml of YePS liquid medium at 28°C with shaking at 200 rpm for 24 h. Aliquots of cultured sporidia were then diluted with fresh YePS liquid medium, the cell density was adjusted to 10^5^ cells per ml, and samples were then cultured for another 48 h under the same conditions. The OD 600 was measured with a spectrophotometer (NanoDrop 2000C) every 6 h to monitor the yeast-like (budding) growth of the wild-type, deletion mutant, or complementary mutant strains.

### Nucleic Acid Manipulation

Fungal genomic DNA was extracted using a modified CTAB method (Shen et al., 2006). The PCR amplification was performed using Phanta High-Fidelity DNA Polymerase (Vazyme, P505). Purification of DNA fragments was conducted using a FastPure Gel DNA Extraction Mini Kit (Vazyme, DC301). Total RNA was extracted with TRIzol (Vazyme, R401), and HISCRIPT III RT SuperMix (Vazyme, R323) was used for cDNA synthesis. A NanoDrop ND-1000 (Thermo Fischer Scientific, Wilmington, DE, United States) was used for measuring the concentration and purity.

### Construction of *SsCI72380* Gene Knockout and Complementary Mutants

The construction of two fragments for the replacement of the *SsCI72380* gene by the *Hpt* (encodes a phosphotransferase conferring hygromycin resistance) gene was based on previous methods (Chakraborty et al., 1991; Yang and Chung, 2012; Li et al., 2014a; Chang et al., 2018). The flanking DNA of the *SsCI72380* gene was PCR-amplified using wild-type *S. scitamineum* genomic DNA (*Ss16^+^* and *Ss16^−^*). The *Hpt* gene in plasmid pDAN was the template. Gene knockout mutants were obtained by polyethylene glycol (PEG)-mediated protoplast transformation (Cai et al., 2020). The construction process of the deletion mutants is shown in Supplementary Figure S1A. The primer design of the amplified products was derived from the genome sequence LK056684.1 in NCBI. All primers involved in the construction and validation of the knockout mutants are indicated in Table 1.

**Table 1:** Primers used in this study.

Name	Primer sequences (5'-3')	Description
*SsCI72380*-LB-F	CGTCGAAGCGCTCAAGTCATC	
*SsCI72380*-LB-R	GTCGTGACTGGGAAAACCCTG AGATCAGGTTGACGGTGAGGG	
*SsCI72380*-RB-F	GGTCATAGCTGTTTCCTGTGTGAATGATGAAGGGATCCGCCAGC	
*SsCI72380*-RB-R	GCCTTTTGGATATGCCCTCGC	Deletion construction
Hpt-LB-F	CAGGGTTTTCCCAGTCACGAC	
Hpt-LB-226	GGTCAAGACCAATGCGGAGC	
Hpt-RB-225	GCAAGACCTGCCTGAAACCG	
Hpt-RB-R	TCACACAGGAAACAGCTATGACC	
*SSCI72380*-JC-F	GCGTTTCGTAGTCCAAGTCCCG	PCR verification
*SSCI72380*-JC-R	GGACTCGACAAGTCGTTCGCAC	
72380COM-F	ATCCAAGCTCAAGCTAAGCTTCGTCGAAGCGCTCAAGTCA	
72380COM-R	CAGCAAGATCTAATCAAGCTTGCCTTTTGGATATGCCCTCG	Complementation construction
COM-HPT-LB-F	GCGCGCGTAATACGACTCAC	
Zeocin-R	GAAGTGCACGCAGTTGCCG	
Situ-F	CTCCGTGTTGATGCTGGGAC	
COM-HPT-RB-R	CGAGCATTCACTAGGCAACCA	
Zeocin-JC-F	CGAGGTGGTTGCCCGTGTTT	
Zeocin-JC-R	CGGAAGTTCGTGGACACGAC	PCR verification
*SSCI72380*-qF	TGATCTCTGGTCGACTAGCT	
*SSCI72380*-qR	CCAGGATATCGTTGCCAAAC	qRT-PCR
Actin-qF	ACAGGACGGCCTGGATAG	
Actin-qR	TCACCAACTGGGACGACA	

The complementation of the *SsCI72380* gene followed a previous strategy (Chang et al., 2018). The complemented gene not only carries the hygromycin homologous fragment to replace the hygromycin fragment in the knocked out mutants but also carries the zeocin resistance marker gene to screen the complements. The construction process of the complementary mutants is shown in Supplementary Figure S1B. Complementary mutants were obtained by PEG-mediated protoplast methods. All primers involved in the construction and validation of the complementary mutants are listed in Table 1.

### Sterol 14α-Demethylase (CYP51) Activity Assay

We assessed the effect of the *SsCI72380* gene on cytochrome P450 sterol 14 alpha-demethylase (CYP51) activity every 12 h over a period of 48 h under haploid sporidia culture conditions (YePS, 28°C, 200 rpm) based on the determination of CYP51 activity. CYP51 activity determination was carried out in accordance with the instructions of the ELISA kit (Shanghai mlbio Enzyme-linked Biotechnology).

### Determination of Ergosterol Content and Lanosterol Content

The determination method referred to Zhang et al. (2009), with slight modifications. A total of 0.5 g sporidia colonies of the wild-type (*Ss16^+^* and *Ss16^−^*), deletion mutant (*ΔSs72380^+^* and *ΔSs72380^−^*), or complementary mutant (*COM72380^+^* and *COM72380^−^*) collected from YePS liquid medium (cultured at 28°C for 48 h) were saponified by 10 ml saponification solution (50% KOH solution: absolute ethanol = 2:3) in a water bath of 88°C for 3 h. Then the products were added to petroleum ether for extraction. After evaporation drying, samples were dissolved in chloroform. Using ergosterol as an external standard, gas chromatography (GC) was used to determine the ergosterol content and lanosterol content (mg/g). Chromatographic determination conditions: Shimadzu gas chromatograph (GCMS-QP2020); capillary column DB-5 (25.0 m × 0.25 mm × 0.25 μm); temperature program: 195°C, 3 min; rise to 300°C with 5.5°C per min; injector temperature: 280°C; sample volume: 1 μl. Each group of experiments was repeated three times, and each sample was injected twice.

### Determination of Conductivity

The conductivity measurement method was performed as reported by Duan et al. (2013). Haploid sporidia colonies (0.1 g) of wild-type (*Ss16^+^* and *Ss16^−^*), deletion mutant (*ΔSs72380^+^* and *ΔSs72380^−^*), or the complementary mutant (*COM72380^+^* and *COM72380^−^*) were scraped and placed into 50 ml of sterile ddH_2_O and cultured at 28°C with shaking at 200 rpm for 2 h. Around 15 ml of each were absorbed separately and centrifuged at a low speed, and the electrical conductivity of the upper liquid (R1) was determined by a conductivity meter (DDS-307A, Shanghai Leici). The remaining spore liquid was boiled in a 100°C water bath for 10 min and then centrifuged at a low speed, and 15 ml was removed to measure the conductivity of the upper liquid (R2). Relative conductivity (%) = (R1/R2) × 100%. Three replicates were set for each sample.

### Analysis of Gene Expression

We assessed the transcriptional profile of the *SsCI72380* gene every 12 h over a period of 72 h under haploid and mating conditions as well as during the infection process (after inoculation of sugarcane plants of the smut susceptible variety “ROC22”) using quantitative real-time PCR (qRT-PCR). For the qRT-PCR, we used a ChamQ Universal SYBR quantitative PCR (qPCR) Master Mix (Vazyme, Q711), and the reaction was run on a Real-Time PCR System (CFX96, BioRad). Relative expression values were calculated with the 2^-ΔΔCt^ method using *ACTIN* as an internal control (Livak and Schmittgen, 2001). Three biological repeats each containing three technical replicas for each sample were performed. The primers used in this study are listed in Table 1.

### Assay of the Pathogenicity of Knockout and Complement Mutants of the *SsCI72380* Gene

Sporidial colonies of the wild-type (*Ss16^+^* and *Ss16^−^*), knockout mutant (*ΔSs72380^+^* and *ΔSs72380^−^*), or complementary mutant (*COM72380^+^* and *COM72380^−^*) were inoculated into 50 ml of YePS liquid medium and cultured at 28°C with shaking at 200 rpm for 2 days. Sporidia were collected by centrifugation and washed twice with ddH_2_O, after which they were re-suspended in YePS liquid medium at a final concentration of 2 × 10^9^ spores/ml. Sporidia of opposite mating types were then mixed in equal volumes, after which 200 μl of this mixture was syringe-injected into 4–5 leaves of seedlings of the highly susceptible sugarcane variety ROC22 (20 plants were inoculated in each treatment). A wild-type mixture (*Ss16^+^* and *Ss16^−^*) served as a positive control, and sterile water was used as a negative control. Inoculated plants were kept in a greenhouse for 4 months. Investigation of the occurrence of sugarcane smut began after 1 month. At the end of each investigation, the diseased plants were labeled to avoid repeated investigations, black whip symptoms were covered with plastic bags to prevent the spread of teliospores, and the number of diseased plants and the morbidity were calculated.

### Microscopy

Images were taken using an Axio Observer Z1 microscope (Zeiss, Jena, Germany) equipped with a sCMOS camera (PCO Edge, Kelheim, Germany).

### Statistic Analysis

Data were expressed as the means ± SE. Differences among different treatments were analyzed using GraphPad Prism 8 software (GraphPad, United States).

## Results

### Identification and Characterization of the *SsCI72380* Gene

**Figure 1:**
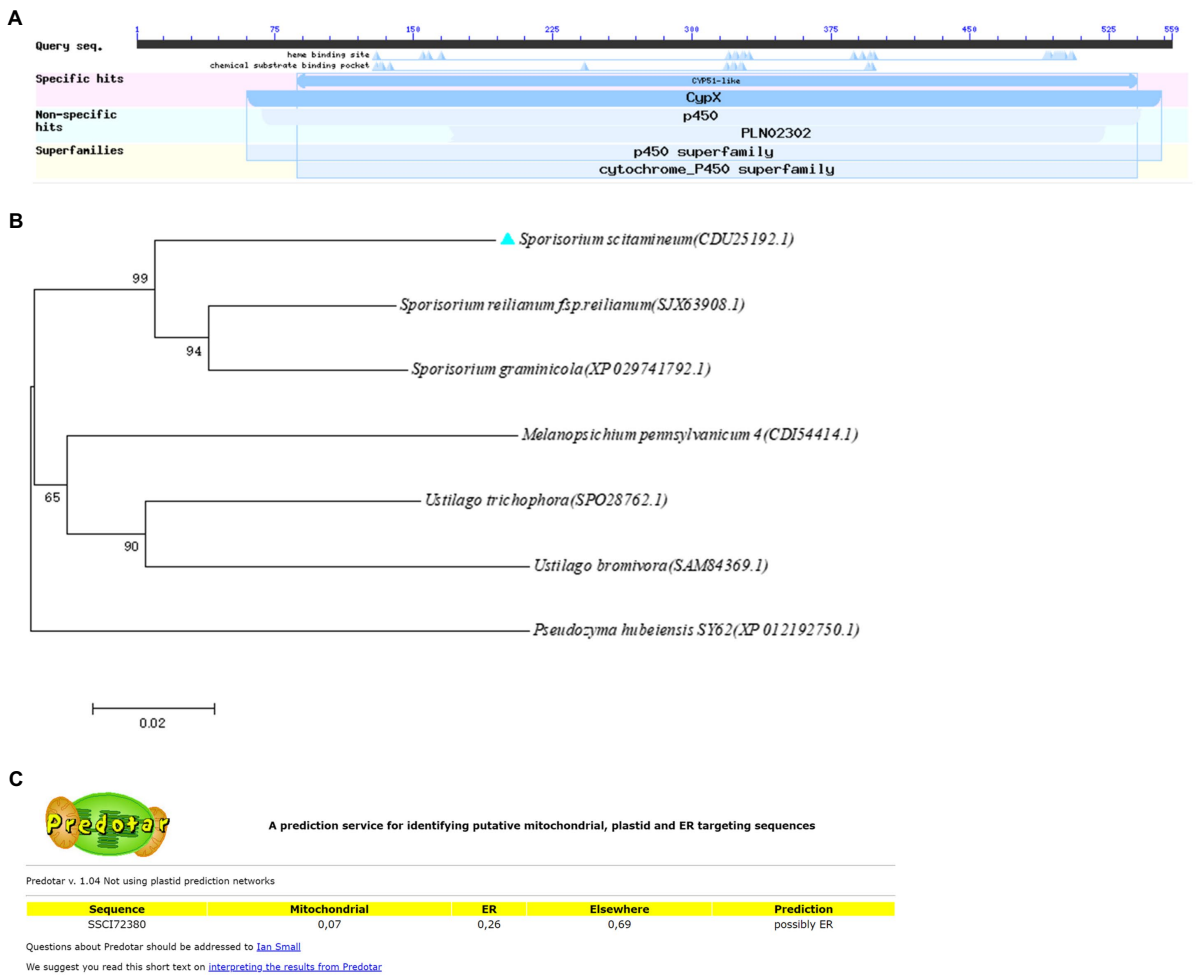
Domain and phylogenetic analysis of *SsCI72380* genes encoding sterol 14α-demethylase in *Sporisorium scitamineum*. **(A)** Predicted protein encoded by the *SSCI72380* gene was sterol 14 alpha-demethylase (p450 family). **(B)** Phylogenetic tree analysis of *SsCI72380* coding proteins; Arabic numerals at the node edge of the tree indicate the degree of credibility of the phylogenetic tree (using 1,000 bootstrap replicates). *Sporisorium scitamineum SsCI72380* is denoted by a blue solid triangle. **(C)** Prediction of the subcellular structural localization of *SsCI72380* gene-coding proteases.

The sterol 14α-demethylase encoding gene *SsCI72380* was previously identified as a significantly (*p* ≤ 0.05) differentially expressed gene in isolates *Ss16* (strong pathogenicity) and *Ss47* (weak pathogenicity) of *S. scitamineum* (Wu et al., 2020). Through NCBI annotation, the gene *SsCI72380* coding protein (NCBI: protein accession no. CDU25192.1) is a sterol 14α-demethylase of the cytochrome P450 family and consists of 559 amino acid residues. The isoelectric point (pI) of the protein was 6.58, the MW was 62.1 kDa, the length of the gene was 1,680 bp, and no intron was present (Figure 1A). Phylogenetic analysis showed that the protein encoded by the gene was highly homologous to *Sporisorium graminisola* conserved hypothetical protein EX895_001104 and *Sporisorium reilianum f.* sp. *reilianum* conserved sterol 14 alpha-demethylase, indicating that the *SsCI72380* gene is highly conserved in smut fungi (Figure 1B). The predicted subcellular structure of the protein was located on the endoplasmic reticulum (Figure 1C).

### Molecular Construction of *SsCI72380* Deletion and Complementary Mutants

**Figure 2:**
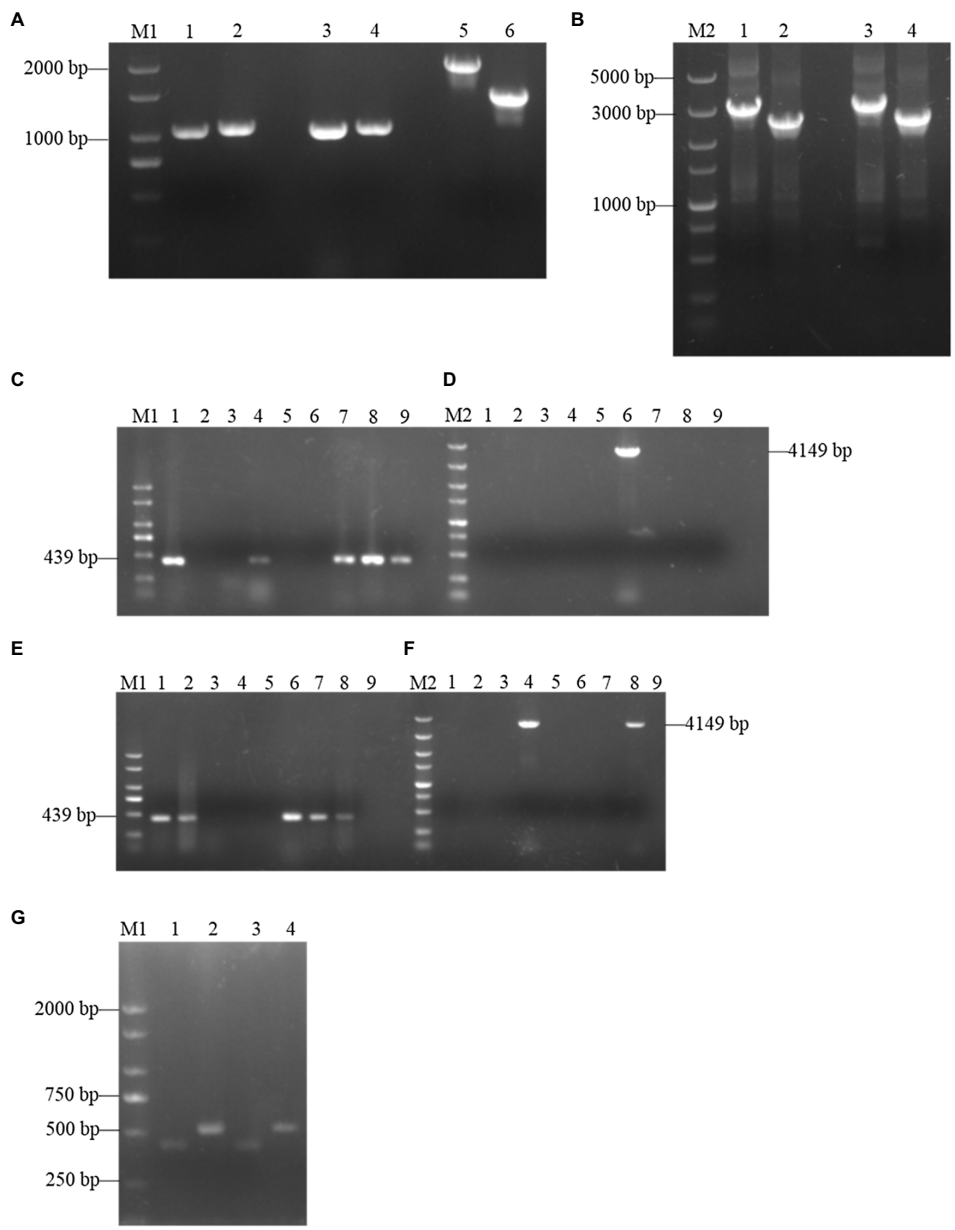
Construction and validation of *SsCI72380* gene knockout and complementary mutants. Lane M1 is the 2,000 marker, and lane M2 is the 5,000 marker. **(A)** PCR amplification. Lanes 1, 3 and 2, 4 represent the right and left borders of the wild-type *Ss16^+^* and *Ss16*^−^, respectively; lanes 5 and 6 represent the two overlapping *HPT* fragments with primer pairs Hpt-LB-F/226 and Hpt-RB-225/R, the band sizes of which were about 2 and 1.5 Kb, respectively. **(B)** Fusion-PCR. Lanes 1 and 3 represent the fusion fragments of the left borders of the wild-type *Ss16^+^* and *Ss16^−^* with the left *HPT* fragments, and lanes 2 and 4 represent the fusion fragments of the right borders of the wild-type *Ss16^+^* and *Ss16^−^* with the right *HPT* fragments, respectively. **(C,D)** The knockout mutant *ΔSs72380^+^* in the *Ss16^+^* background was confirmed by PCR. In **(C,D)**, lanes 2–9 are transformants, lane 1 represents the wild-type. **(C,D)** denote that lane 6 was the deletion mutant as the internal primer pair *SsCI72380*-JC-F/*SsCI72380*-JC-R was unable to produce a 439 bp band, while the external primer pair *SsCI72380*-LB-F/Hpt-RB-R produced a 4,149 bp band corresponding to the inserted *HPT* gene from the deletion mutant. **(E,F)** The knockout mutant *ΔSs72380^−^* in the *Ss16^−^* background was confirmed by PCR. In **(E,F)**, lanes 2–9 are transformants, lane 1 represents the wild-type. In the same way, **(E,F)** denote that lane 4 was the deletion mutant. **(G)** Electrophoretic validation of complementary mutants (*COM72380^+^* and *COM72380^−^*) positive transformants. Lanes 1 and 2 were verified by complementary mutant electrophoresis in *ΔSs72380^+^* background, lanes 3 and 4 were verified by complementary mutant electrophoresis in *ΔSs72380^−^* background, lanes 1 and 3 were the *SsCI72380* target gene fragment, and lanes 2 and 4 were the target gene fragment of zeocin.

To further investigate the functions of *SsCI72380*, gene deletion and complementary strains were constructed as described in the Materials and Methods. Using wild-type *S. scitamineum* genomic DNA (*Ss16^+^* and *Ss16^−^*) as template, each fragment was amplified by PCR with the primer pairs *SsCI72380*-LB-F/R and *SsCI72380*-RB-F/R. The band sizes were 1,101 and 1,052 bp (Figure 2A), and a fragment fusion (Figure 2B) was used to transform the fragments into *S. scitamineum* wild-type protoplasts with the primer pairs *SsCI72380*-LB-F/Hpt-LB-226 and *SsCI72380*-RB-F/Hpt-RB-R, the band sizes of which were about 3 and 2.5 Kb, respectively. As expected, two deletion mutants (*ΔSs72380^+^* and *ΔSs72380^−^*) were obtained. The *SsCI72380* deletion mutants were confirmed with PCR using the primer pairs *SsCI72380*-JC-F/*SsCI72380*-JC-R and *SsCI72380*-LB-F/Hpt-RB-R. Use of the primer pair *SsCI72380*-JC-F/*SsCI72380*-JC-R resulted in a 439 bp band from the *SsCI72380* gene in the wild-type (*Ss16^+^* and *Ss16^−^*), but not in the deletion mutants, while the primer pair *SsCI72380*-LB-F/Hpt-RB-R produced a 4,149 bp band corresponding to the inserted *HPT* gene from the deletion mutants (Figures 2C–F). The *SsCI72380* complementary mutants (*COM72380^+^* and *COM72380^−^*) were confirmed with PCR using the primer pairs Zeocin-JC-F/Zeocin-JC-R (583 bp) and *SsCI72380*-JC-F/*SsCI72380*-JC-R (439 bp; Figure 2G).

### Morphology, Growth, and Mating/Filamentation of *S. scitamineum*

**Figure 3:**
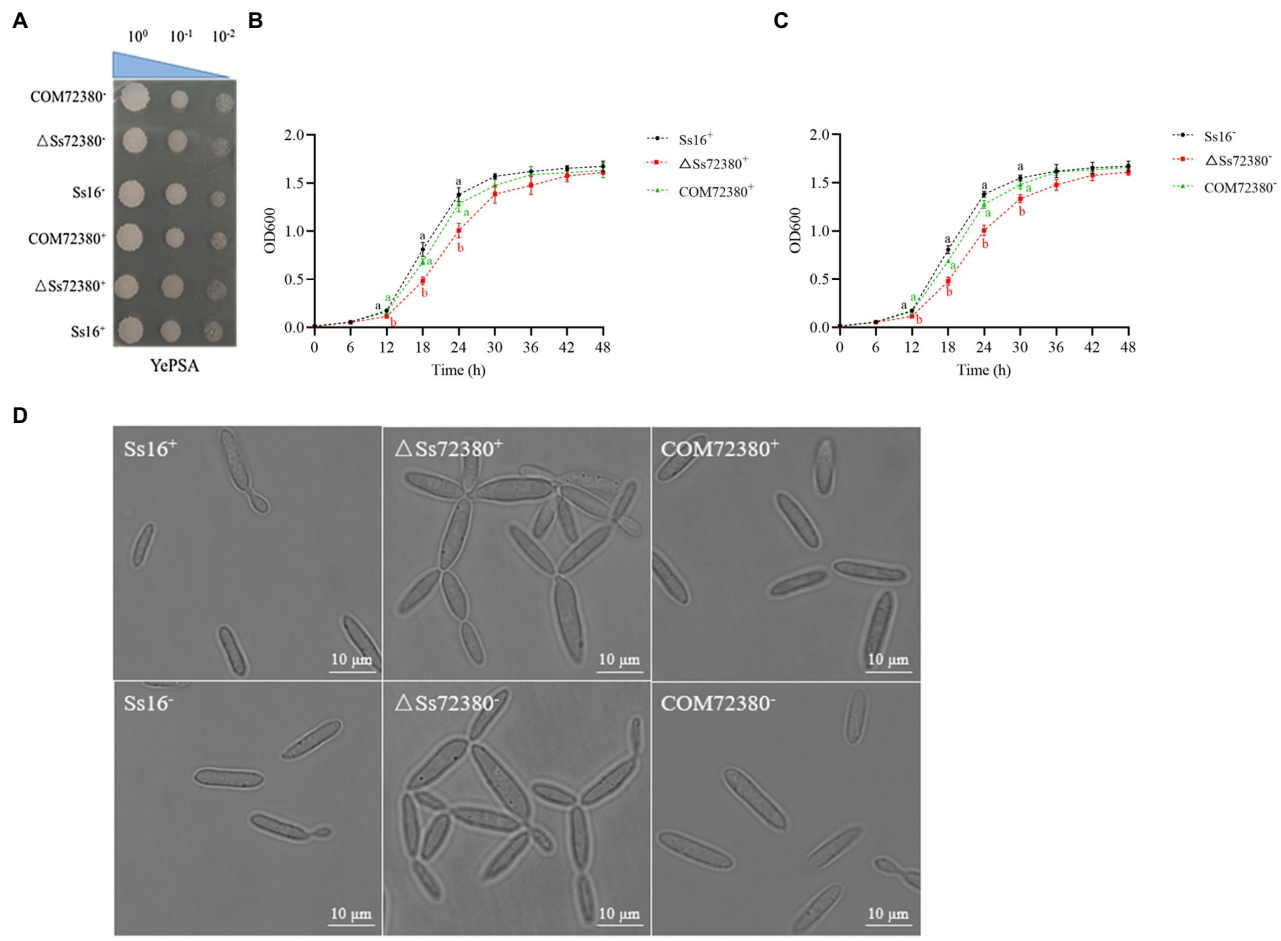
Effects of the *SsCI72380* gene on the haploid phenotype of *S. scitamineum*. **(A)** Haploid colonies of wild-types (*Ss16^+^* and *Ss16^−^*), knockout mutants (*ΔSs72380^+^* and *ΔSs72380^−^*), and complementary mutants (*COM72380^+^* and *COM72380^−^*) were spotted on YePSA plates and incubated at 28°C for 48 h. **(B,C)** Haploid growth curve, each sample had three replicates. Bars indicate the SE. Different lowercase letters represent a difference at the 0.05 level. **(D)** Microscopic images of sporidia of WT, deletion mutants, and complementary mutants (YePS liquid medium, 28°C, 200 rpm, 48 h, observed under 100× oil immersion microscopy).

It was observed that the haploid colony morphology of the *SsCI72380* gene knockout mutant (*ΔSs72380^+^* and *ΔSs72380^−^*) on the YePSA plates was not different from that of the wild-type strains (*Ss16^+^* and *Ss16^−^*) and complementary mutants (*COM72380^+^* and *COM72380^−^*; Figure 3A). In contrast, the growth rate of the knockout mutants (*ΔSs72380^+^* and *ΔSs72380^−^*) was found to be lower than that of the wild-type strains (*Ss16^+^* and *Ss16^−^*), and the growth rate of the complementary mutants (*COM72380^+^* and *COM72380^−^*) basically returned to that of the wild-type strains (Figures 3B,C).

The morphological changes in the haploid spores of the *SsCI72380* gene knockout mutant (*ΔSs72380^+^* and *ΔSs72380^−^*) were observed under a microscope. Most of the knockout mutants were pseudomycelium-like, while the wild-type (*Ss16^+^* and *Ss16^−^*) and complementary mutants (*COM72380^+^* and *COM72380^−^*) were long oval rods or two connected long rods at the mitotic stage (Figure 3D).

**Figure 4:**
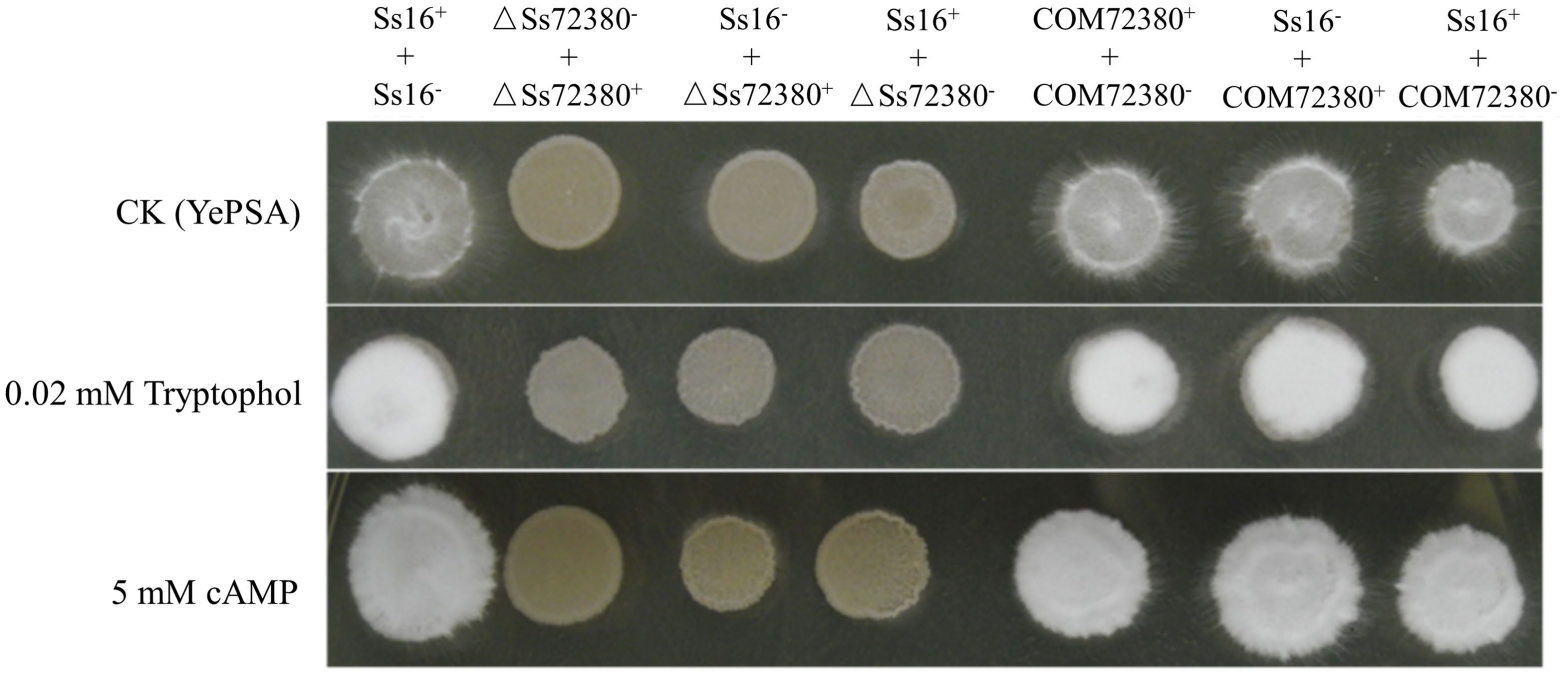
Effect of the *SsCI72380* gene on the sexual mating of *S. scitamineum*. Sexual mating was assessed on YePSA plates supplemented with cAMP (5 mM) or tryptophol (0.02 mM). Photographs were taken 42 h after inoculation. The control was an untreated culture.

As shown in Figure 4, based on the determination of the sexual mating ability of *S. scitamineum* on YePSA plates, it was found that the sexual mating ability between the two knockout mutants (*ΔSs72380^+^* and *ΔSs72380^−^*) was significantly weakened, and almost no white villous hyphae were produced. The sexual mating ability between the knockout mutant and the wild-type (*Ss16^+^* and *ΔSs72380^−^* or *Ss16^−^* and *ΔSs72380^+^*) was also significantly weaker than that between the wild-type (*Ss16^+^* and *Ss16^−^*), and the sexual mating ability between the complementing mutants (*COM72380^+^* and *COM72380^−^*) almost recovered to that between the wild-type. The addition of previously reported small-molecule signaling substances (tryptophol or cAMP) involved in the sexual mating of *S. scitamineum* (Deng et al., 2018b; Wang et al., 2019) promoted the sexual mating ability of the wild-type and complemented mutants, but could not restore the sexual mating defect of the knockout mutants.

### Effect of the *SsCI72380* Gene on the CYP51 Activity of *S. scitamineum*

**Figure 5:**
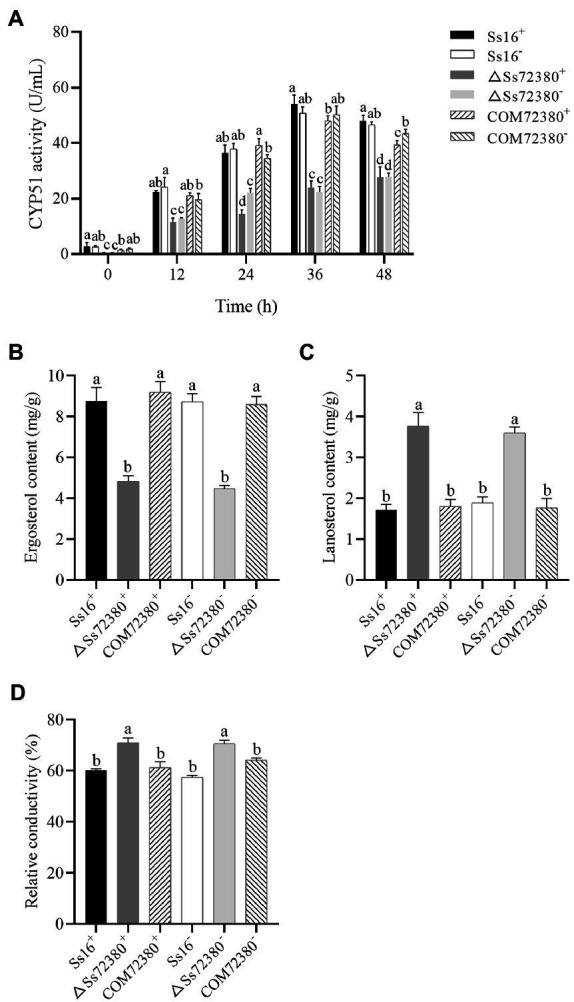
Effect of the *SsCI72380* gene on cytochrome P450 sterol 14 alpha-demethylase (CYP51) activity **(A)**, ergosterol content **(B)**, lanosterol content **(C)**, and the conductivity **(D)** in *S. scitamineum*. Bars indicate the SE derived from three independent replicates. In the same group, different lowercase letters represent a difference at the 0.05 level.

In the sporidia growth process of *S. scitamineum*, the CYP51 activity of the knockout mutants (*ΔSs72380^+^* and *ΔSs72380^−^*) was always significantly lower than that of the wild-type (*Ss16^+^* and *Ss16^−^*) and complemented mutants (*COM72380^+^* and *COM72380^−^*), while the CYP51 activity of the complemented mutants (*COM72380^+^* and *COM72380^−^*) was almost equal to that of the wild-type (*Ss16^+^* and *Ss16^−^*; Figure 5A).

### *SsCI72380* Genes Involved in Ergosterol Biosynthesis of the Cell Membrane in *S. scitamineum*

The ergosterol content of the *SsCI72380* knockout mutants (*ΔSs72380^+^* and *ΔSs72380^−^*) was significantly lower than that of the wild-type (*Ss16^+^* and *Ss16^−^*) and complemented mutants (*COM72380^+^* and *COM72380^−^*), while the ergosterol content of the complemented mutants (*COM72380^+^* and *COM72380^−^*) was almost the same as that of the wild-type (*Ss16^+^* and *Ss16^−^*), in addition, the content of lanosterol, CYP51 substrate, showed the opposite trend, indicating that the biosynthesis of ergosterol in *S. scitamineum* requires the participation of the *SsCI72380* gene (Figures 5B–C).

### Comparative Analysis of the Conductivity of a Sporidial Solution of *S. scitamineum*

The conductivity of the *SsCI72380* gene knockout mutants (*ΔSs72380^+^* and *ΔSs72380^−^*) was significantly higher than that of the wild-type (*Ss16^+^* and *Ss16^−^*) and complementary mutants (*COM72380^+^* and *COM72380^−^*), and the conductivity of the complementary mutants (*COM72380^+^* and *COM72380^−^*) was almost the same as that of the wild-type (*Ss16^+^* and *Ss16^−^*), indicating that the *SsCI72380* gene is involved in the regulation of cell membrane stability in *S. scitamineum* (Figure 5D).

### Assessment of Stress Tolerance

**Figure 6:**
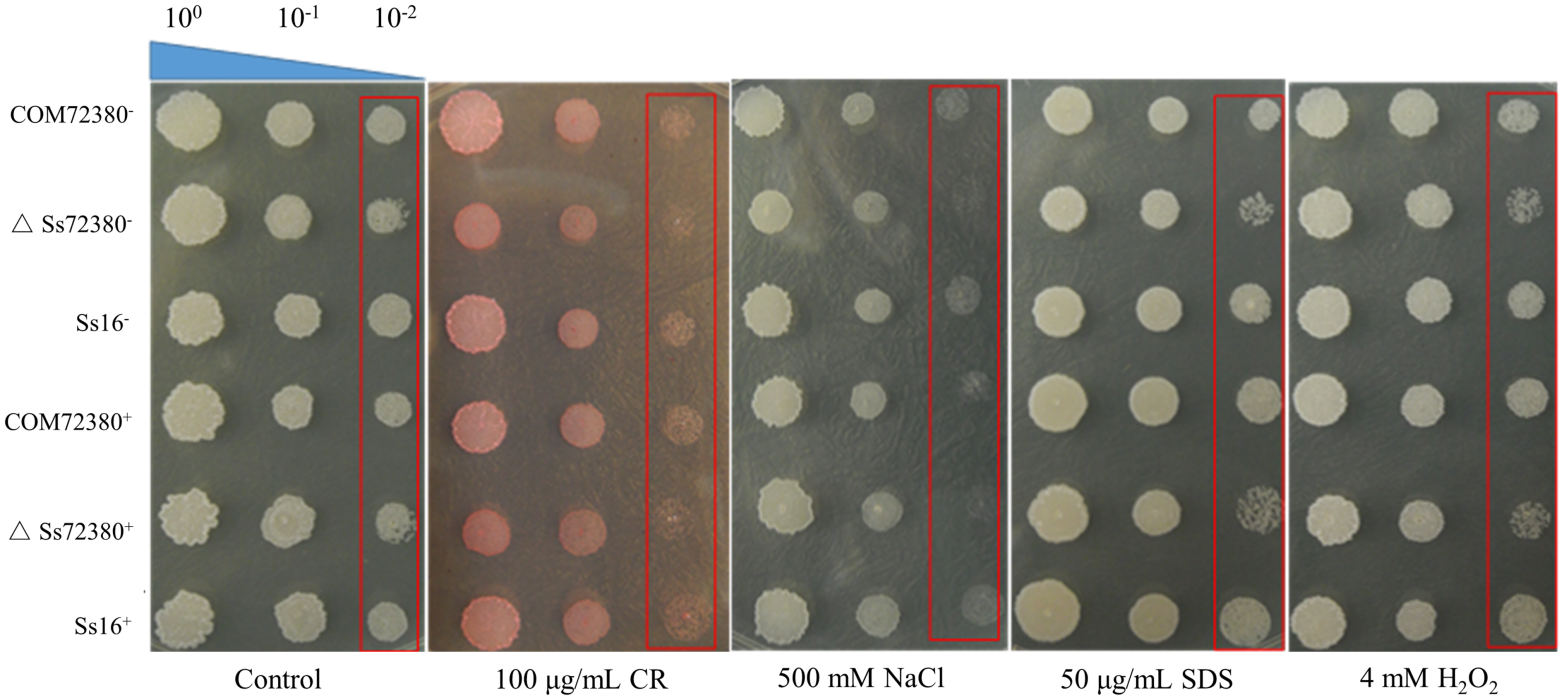
Effect of the *SsCI72380* gene on abiotic stress in *S. scitamineum*. Serially diluted cells of WT (*Ss16^+^* and *Ss16^−^*), deletion mutants (*ΔSs16^+^* and *ΔSs16^−^*), or complementary mutants (*COM72380^+^* and *COM72380^−^*) were spotted onto YePSA plates supplemented with H_2_O_2_ (4 mM), NaCl (500 mM), SDS (50 μg/ml), or Congo red (CR; 100 μg/ml). Samples were incubated at 28°C for 48 h before examination.

We examined the tolerance toward various stressful conditions in the wild-type and mutant sporidia, including cell wall stress (SDS or Congo red), hyperosmotic stress (NaCl), and oxidative stress (H_2_O_2_; Figure 6). The growth rate of the *SsCI72380* gene knockout mutants (*ΔSs72380^+^* and *ΔSs72380^−^*) in YePSA medium was slower than that of the wild-type (*Ss16^+^* and *Ss16^−^*) and complemented mutants (*COM72380^+^* and *COM72380^−^*). On YePSA medium supplemented with Congo red, SDS, NaCl, and H_2_O_2_, the growth rate of the *SsCI72380* gene knockout mutants was further slower than that of the wild-type and complemented mutants (especially at low concentrations), and the growth rate of the complemented mutants was basically the same as that of the wild-type (Figure 6). The results showed that the *SsCI72380* gene was involved in physiological processes such as hyperosmotic, oxidative, or cell wall integrity (CWI) stress responses in *S. scitamineum*.

### *SsCI72380* Gene Expression Level

**Figure 7:**
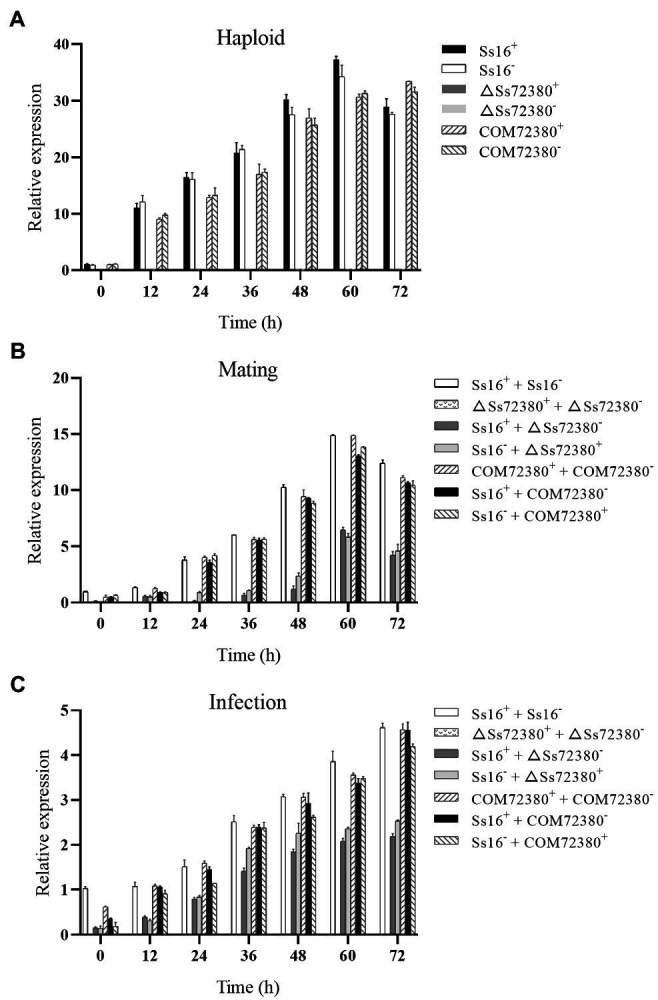
Expression analysis of *SsCI72380* during various stages. **(A)** Gene expression levels under sporidial growth. **(B)** Gene expression levels under sexual mating conditions. **(C)** Gene expression levels under sugarcane bud infection. *SsCI72380* expression at 0 h was normalized to one. Bars indicate the SE. The analyses were repeated three times.

The *SsCI72380* gene expression of the tested strains was determined by qRT-PCR, and the results are shown in Figure 7. During the growth of the haploid sporidia of *S. scitamineum*, the expression of the *SsCI72380* gene in the wild-type (*Ss16^+^* and *Ss16^−^*) and complementary mutants (*COM72380^+^* and *COM72380^−^*) increased with the growth of the sporidia, while the expression of the *SsCI72380* gene in the knockout mutants (*ΔSs72380^+^* and *ΔSs72380^−^*) was undetectable (Figure 7A). In the sexual mating stage, the expression of the *SsCI72380* gene first increased and then decreased with culture time, and the maximal expression occurred at around 60 h. The expression level of the *SsCI72380* gene between the wild-type (*Ss16^+^* and *Ss16^−^*) and complementing mutants (*COM72380^+^* and *COM72380^−^*) or between the wild-type and complementing mutants (*Ss16^+^* and *COM72380^−^* or *COM72380^+^* and *Ss16^−^*) was significantly higher than that between the wild-type and knockout mutants (*Ss16^+^* and *ΔSs72380^−^* or *Ss16^−^* and *ΔSs72380^+^*) or between the complementary mutants and knockout mutants (*COM72380^+^* and *ΔSs72380^−^* or *COM72380^−^* and *ΔSs72380^+^*), while the expression level of the *SsCI72380* gene was not detected between the knockout mutants (*ΔSs72380^+^* and *ΔSs72380^−^*; Figure 7B). During the process of sugarcane bud infection, the expression of the *SsCI72380* gene was similar to that in the process of sexual mating (Figure 7C).

### The *SsCI72380* Gene Is Required for the Full Pathogenicity of *S. scitamineum*

**Figure 8:**
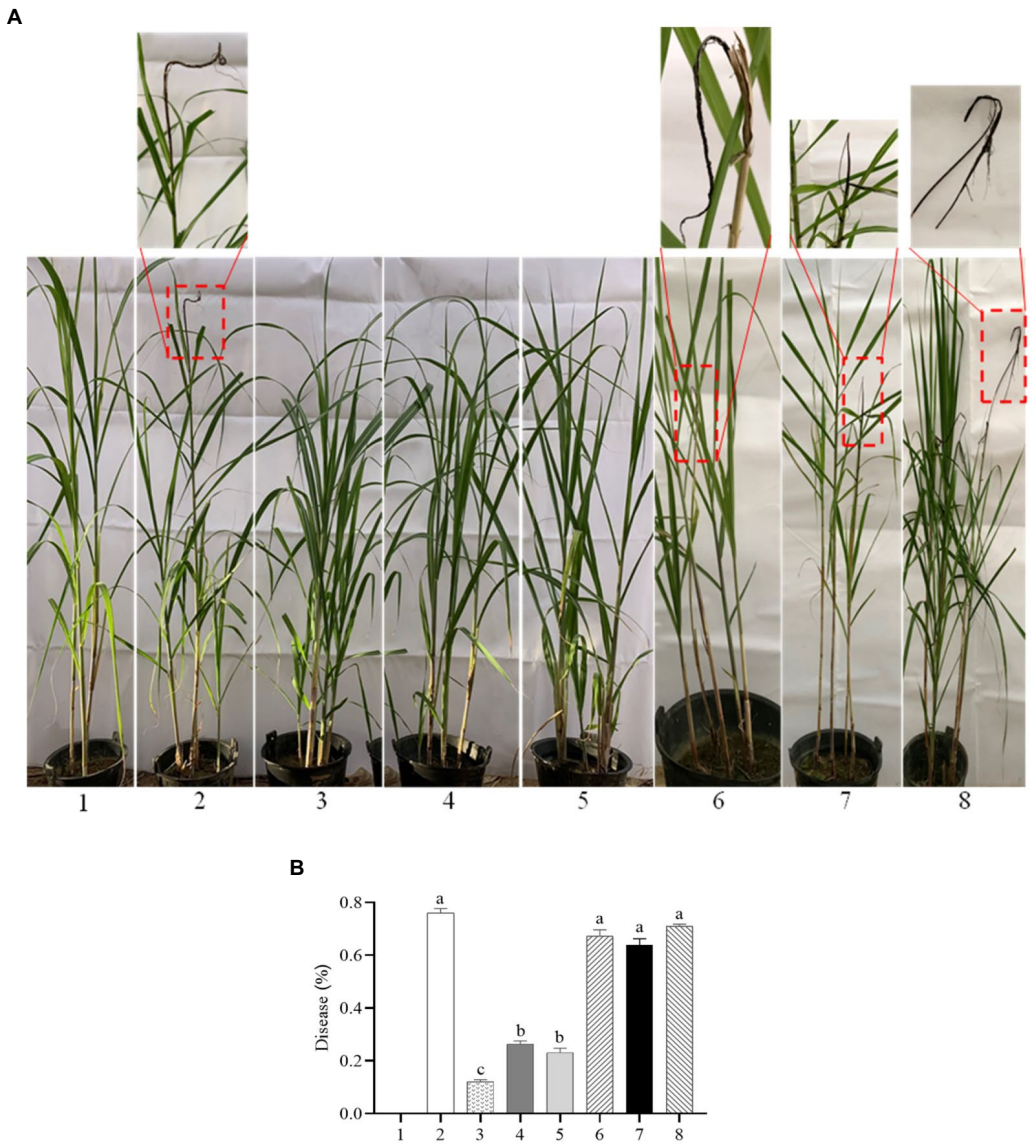
*SsCI72380* gene involved in the pathogenicity of *S. scitamineum*. **(A)** Symptoms of smut whip. **(B)** Incidence. Numbers indicate: sterile water (1), *Ss16^+^* + *Ss16^−^* (2), *ΔSsCI72380^+^* + *ΔSsCI72380^−^* (3), *Ss16^+^* + *ΔSsCI72380^−^* (4), *Ss16^−^* + *ΔSsCI72380^+^* (5), *COM72380^+^* + *COM72380^−^* (6), *Ss16^+^* + *COM72380^−^* (7), and *Ss16^−^* + *COM72380^+^* (8) inoculation, respectively. Bars indicate the SE. Different lowercase letters represent a difference at the 0.05 level.

To test whether *SsCI72380* is required for *S. scitamineum* pathogenicity, the highly susceptible sugarcane variety ROC22 was inoculated by injection with mixed fungal sporidia of different combinations (of opposite mating types) as follows: *∆SsCI72380^+^* + *∆SsCI72380^−^*, *∆SsCI72380^−^* + *Ss16^+^*, *∆SsCI72380^+^* + *Ss16^−^*, *COM72380^+^* + *COM72380^−^*, *Ss16^+^* + *COM72380^−^*, and *Ss16^−^* + *COM72380^+^*, as well as with the wild-type *Ss16^+^* + *Ss16^−^* combination as a positive, and sterile water was used as a negative control. The combinations containing knockout mutants (*∆SsCI72380^+^* + *∆SsCI72380^−^*, *∆SsCI72380^−^* + *Ss16^+^*, and *∆SsCI72380^+^* + *Ss16^−^*) displayed significantly reduced pathogenicity, with only 12, 26, and 23% of the infected seedlings showing black whip symptoms, respectively. In contrast, the combinations without knockout mutants (*COM72380^+^* + *COM72380^−^*, *Ss16^+^* + *COM72380^−^*, and *Ss16^−^* + *COM72380^+^*) showed significantly high incidence rates, and the wild-type *Ss16^+^* + *Ss16^−^* mixture led to 76% of the infected seedlings having black whip symptoms (Figure 8). Therefore, we conclude that *SsCI72380* is required for the full pathogenicity of *S. scitamineum*.

## Discussion

In this study, based on the transcriptome sequencing data of different pathogenic isolates of *S. scitamineum* (Wu et al., 2020), a gene *SsCI72380* encoding sterol 14α-demethylase was screened from the most enriched pathways in the Kyoto Encyclopedia of Genes and Genomes (KEGG) database. The phylogenetic tree showed that the protein encoded by the gene was highly conserved among smut fungi. In addition, the subcellular localization predicted that the protein encoded by the gene might be located on the endoplasmic reticulum (The prediction results of subcellular localization by different tools are inconsistent, and some predictions are located on the plasma membrane, which needs further research). Among the biosynthetic pathways of ergosterol in biological *S. cerevisiae*, sterol 14α-demethylase is involved in the synthesis of ergosterol (Jordá and Puig, 2020). Ergosterol is a common sterol compound found in fungi that is mainly involved in the biological processes of fungal cell membrane fluidity, structural maintenance, sexual development, cell viability, and growth (Alvarez et al., 2007; Jin et al., 2008; Abe et al., 2009; Martínez et al., 2011). In *Aspergillus fumigatus*, there are two homologous genes of the yeast ERG11 gene (encoding sterol 14α-demethylase), namely erg11a and erg11b. Deletion of erg11b reduces ergosterol content (Hu et al., 2007). Sterol 14α-demethylase is a key enzyme in ergosterol biosynthesis. The synthesis of ergosterol is partially blocked, which will destroy the membrane structure of fungi and reduce the growth rate of fungi. In yeast, deletion of the gene encoding the enzyme can change the cell proliferation status and decrease the growth rate (Odds et al., 2003; Chen et al., 2009). Gene *ERG11* knockout mutants in *Candida albicans* showed obvious filament defects, morphological changes in the hyphae, and decreased pathogenicity (Wu et al., 2018). However, the genes encoding sterol 14α-demethylase and their biological functions in *S. scitamineum* have not been reported.

In this study, the knockout mutants and complementary mutants of gene *SsCI72380* encoding sterol 14α-demethylase were obtained from *S. scitamineum* by gene knockout and gene complementation techniques. The CYP51 activity, electrical conductivity, ergosterol content, and lanosterol content in the wild-type strains, knockout mutants, and complementary mutants were determined. The CYP51 activity of the knockout mutants decreased much more than the wild-type strains. However, the CYP51 activity did not decrease to zero in the knockout mutants. It is speculated that there should be additional genes encoding CYP51 involved in the regulation of CYP51 activity. In fact, through NCBI annotation, we found another gene (GenBank accession no. LK056650.1) encoding CYP51 in *S. scitamineum*. There are similar phenomena in some fungi. For example, there are two genes encoding CYP51 in *A. fumigatus* and three genes encoding CYP51 in *Fusarium graminearum* (Hu et al., 2007; Fan et al., 2013); the electrical conductivity of the knockout mutants increased significantly compared with that of the wild-type strains; the content of ergosterol decreased significantly compared with that of the wild-type strains. The ergosterol content of the mutants was not zero, possibly due to the existence of additional genes encoding CYP51. Similarly, *A. fumigatus* exists two genes, *ERG11A* and *ERG11B*, encoding CYP51. Deletion of *ERG11A* has no effect on ergosterol content, while deletion of *ERG11B* significantly reduces ergosterol content. The double deletion of *ERG11A* and *ERG11B* leads to the death of the fungus (Hu et al., 2007); and the content of lanosterol, the substrate of CYP51, showed the opposite change. The CYP51 activity of the knockout mutants decreased significantly, resulting in the accumulation of the lanosterol. There is a similar phenomenon in *S. cerevisiae*. In the knockout mutant of the gene *ERG11* encoding CYP51, the content of lanosterol is significantly higher than that of the wild-type (Chen et al., 2009). However, the CYP51 activity, electrical conductivity, ergosterol contents, and lanosterol contents of the complementary mutants were consistent with those of the wild-type strains. It is speculated that the deletion of this gene does not inhibit ergosterol biosynthesis, it reduces ergosterol production in *S. scitamineum* and partially affect the integrity of membrane structure and function in *S. scitamineum*. Our findings corroborate previous reports that the deletion of the sterol 14α-demethylase gene in some fungi partially blocks ergosterol synthesis and leads to the destruction of fungal cell membrane structure (Hu et al., 2007).

The development of fungal pathogenicity is closely related to the growth and morphology of the spores. *Aflcla4* knockout mutants of the pathogenic filamentous fungus *Aspergillus flavus* indicated abnormal branching during mycelial growth and significantly reduced conidia production, resulting in a significant decline in pathogenicity and virulence (Qin et al., 2020). The deletion mutant of *CfMKK1* (encoding mitogen-activated protein kinase) of *Colletotrichum fructicola* significantly reduced the growth rate of the spores, failed to form conidiospore appressorium, and reduced the pathogenicity (Xiao and Li, 2020). The knockout mutants of gene *Cla4* (encoding PAK family kinases) in *Ustilago maydis* were forked in haploid morphology, which reduced the sexual compatibility and pathogenicity of *U. maydis* (Leveleki et al., 2004). In this study, we found that the growth rate of the knockout mutants of gene *SsCI72380* in *S. scitamineum* was slower than that of the wild-type strains. Under abiotic stresses such as H_2_O_2_, NaCl, SDS, and Congo red, the growth rate of the knockout mutants was even lower than that of the wild-type strains, and the growth rate of the complementary mutants was basically the same as that of the wild-type strains. Through *S. scitamineum* haploid morphological observation and comparison, it was found that the haploid spores of the knockout mutant showed a series of connected forked branches, while the spores of the wild-type and the complementary mutant showed a single short rod or two short rod cells connected together due to the division stage. A pathogenicity test showed that the incidence rate of sugarcane smut in the mixed inoculation group containing *SsCI72380* gene knockout mutants (*∆SsCI72380^+^* + *∆SsCI72380^−^*, *Ss16^+^* + *∆SsCI72380^−^*, or *Ss16^−^* + *∆SsCI72380^+^*) was significantly lower than that in the wild-type mixed inoculation group (*Ss16^+^* + *Ss16^−^*). Our findings indicate that the deletion of gene *SsCI72380* affects the growth rate, morphology, and pathogenicity of *S. scitamineum*. This is similar to previous studies on pathogenic genes in *A. flavus* (Qin et al., 2020), *C. fructicola* (Xiao and Li, 2020), and *U. maydis* (Leveleki et al., 2004) in pathogenic filamentous fungus.

As a dimorphic fungus, the pathogenicity of *S. scitamineum* is closely related to sexual mating and the formation of dikaryotic hypha (Que et al., 2014). Previous studies have focused on signaling pathways and small-molecule signaling substances, such as tryptophol or cAMP, which are associated with sexual mating in *S. scitamineum* (Chang et al., 2018; Wang et al., 2019). In this study, the *SsCI72380* knockout mutants showed significantly decreased sexual mating ability, and their sexual mating ability was not restored after the addition of small-molecule signaling substances such as tryptophol or cAMP. It is suggested that the gene *SsCI72380* may not be involved in the synthesis or transport of small-molecule signaling substances related to sexual mating in *S. scitamineum*. The gene *SsCI72380* is mainly involved in the biosynthesis of ergosterol in the cell membrane, regulates the integrity of cell membrane structure and function, and affects the growth rate and morphological changes of *S. scitamineum*, thus weakening its sexual mating ability and pathogenicity development. This differs completely from previous studies on the pathogenic mechanism of *S. scitamineum* based on sexual mating by the synthesis of small-molecule signal substances or the regulation of the signaling pathway (Chang et al., 2018; Deng et al., 2018b; Sun et al., 2019; Wang et al., 2019; Zhang et al., 2019). Therefore, the *SsCI72380* gene found in this study is a new type of pathogenic gene of *S. scitamineum* – a finding that contributes to the elucidation of the pathogenic mechanism of *S. scitamineum*.

In conclusion, PEG-mediated protoplast transformation technology was used to successfully obtain *SsCI72380* knockout mutants and complementary mutants of *S. scitamineum*. Comparative analysis of gene *SsCI72380* expression level, CYP51 activity, ergosterol content, lanosterol content, conductivity, growth rate, spore morphology, and abiotic stress showed that gene *SsCI72380* is mainly involved in ergosterol biosynthesis, regulating the integrity of cell membrane structure and function, and its mutation affects the growth rate and spore morphology, and weakening the sexual mating ability, thus reducing the pathogenicity of *S. scitamineum*. In addition, this study showed that gene *SsCI72380* constitutes a new type of pathogenic gene of *S. scitamineum* that enhances the pathogenicity of *S. scitamineum*.

## Data Availability Statement

The datasets presented in this study can be found in online repositories. The names of the repository/repositories and accession number(s) can be found at: https://www.ncbi.nlm.nih.gov/, MZ004860.

## Author Contributions

WS conceived and designed the experimental plan. HL, YC, and HB performed the experiments. HL, YC, and WS analyzed the data and wrote the manuscript. WS, YC, HL, QD, and JC revised the paper. All authors contributed to the article and approved the submitted version.

## Funding

This work was supported by grants from the Earmarked Fund for National Natural Science Foundation of China (31771861 and 32172063) and Guangdong Provincial Team of Technical System Innovation for Sugarcane Sisal Hemp Industry (2021KJ104-07).

## Conflict of Interest

The authors declare that the research was conducted in the absence of any commercial or financial relationships that could be construed as a potential conflict of interest.

## Publisher’s Note

All claims expressed in this article are solely those of the authors and do not necessarily represent those of their affiliated organizations, or those of the publisher, the editors and the reviewers. Any product that may be evaluated in this article, or claim that may be made by its manufacturer, is not guaranteed or endorsed by the publisher.

## Footnotes

^1^ https://web.expasy.org/compute_pi

^2^ https://www.novopro.cn/tools/translate.html

^3^ https://blast.ncbi.nlm.nih.gov/Blast.cgi?PROGRAM=blastp&PAGE_TYPE=BlastSearch&LINK_LOC=blasthome

^4^ https://urgi.versailles.inra.fr/predotar/

## Supplementary Material

Supplementary Figure S1 The schematic diagram of the mutant construction process. **(A)** The construction process of deletion mutants. **(B)** The construction process of complementary mutants.
