# Supplementary figures and images for: Cytochrome P450 Sterol 14 Alpha-Demethylase Gene SsCI72380 Is Required for Mating/Filamentation and Pathogenicity in Sporisorium scitamineum
Source: Front Microbiol. 2021 Dec 23;12:696117. doi: 10.3389/fmicb.2021.696117 (PMC8733404; doi:10.3389/fmicb.2021.696117)

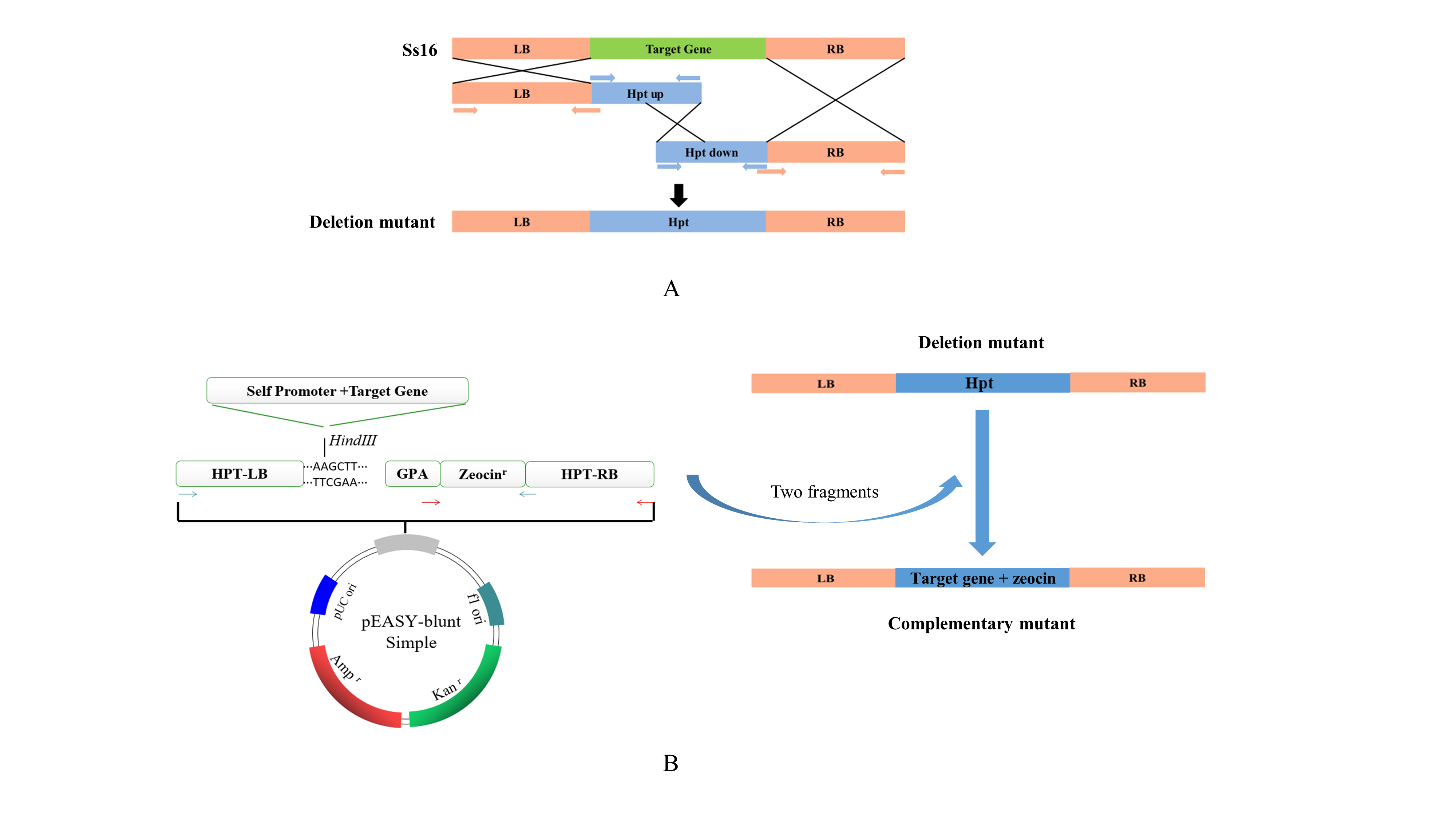

Supplement: Supplementary Figure S1 — The schematic diagram of the mutant construction process. (A) The construction process of deletion mutants. (B) The construction process of complementary mutants. [file Image_1.tif]
